# Patients' Preferences for Participation in Treatment Decision-Making at the End of Life: Qualitative Interviews with Advanced Cancer Patients

**DOI:** 10.1371/journal.pone.0100435

**Published:** 2014-06-25

**Authors:** Linda Brom, H. Roeline W. Pasman, Guy A. M. Widdershoven, Maurice J. D. L. van der Vorst, Jaap C. Reijneveld, Tjeerd J. Postma, Bregje D. Onwuteaka-Philipsen

**Affiliations:** 1 Department of Public and Occupational Health, EMGO Institute for Health and care research, Expertise Center for Palliative Care, VU University Medical Center, Amsterdam, The Netherlands; 2 Department of Medical Humanities, EMGO Institute for Health and care research, VU University Medical Center, Amsterdam, The Netherlands; 3 Department of Medical Oncology, VU University Medical Center, Amsterdam, The Netherlands; 4 Department of Neurology, VU University Medical Center, Amsterdam, The Netherlands; The University of Hong Kong, Hong Kong

## Abstract

**Purpose:**

Patients are often encouraged to participate in treatment decision-making. Most studies on this subject focus on choosing between different curative treatment types. In the last phase of life treatment decisions differ as they often put more emphasis on weighing quantity against quality of life, such as whether or not to start treatment aimed at life prolongation but with the possibility of side effects. This study aimed to obtain insight into cancer patients' preferences and the reasons for patients' preferred role in treatment decision-making at the end of life.

**Methods:**

28 advanced cancer patients were included at the start of their first line treatment. In-depth interviews were held prior to upcoming treatment decisions whether or not to start a life prolonging treatment. The Control Preference Scale was used to start discussing the extent and type of influence patients wanted to have concerning upcoming treatment decision-making. Interviews were audio taped and transcribed.

**Results:**

All patients wanted their physician to participate in the treatment decision-making process. The extent to which patients themselves preferred to participate seemed to depend on how patients saw their own role or assessed their own capabilities for participating in treatment decision-making. Patients foresaw a shift in the preferred level of participation to a more active role depending in the later phase of illness when life prolongation would become more limited and quality of life would become more important.

**Conclusion:**

Patients vary in how much involvement they would like to have in upcoming treatment decision-making. Individual patients' preferences may change in the course of the illness, with a shift to more active participation in the later phases. Communication about patients' expectations, wishes and preferences for participation in upcoming treatment decisions is of great importance. An approach in which these topics are openly discussed would be beneficial.

## Introduction

Patients are encouraged to participate in decisions affecting their own treatment and care. Over the past 40 years, there has been a shift from patients preferring a ‘paternalistic attitude' role from their physician to a more active and decisive role for themselves [1]–[3]. Patients have become better educated and informed about health care issues, but also a change in society's expectations of the appropriate role for physicians has influenced this shift [4]. It is assumed that patients prefer to share decision-making with their physician, and indeed studies have shown that patients prefer a role in which they share the responsibility for the treatment decision with the physician [4]–[8]. However, a considerable number of patients have a preference for a particular type of role, with some preferring an active role for participation in treatment decision-making and others a passive one [9], [10]. Some studies have suggested that increasing patient involvement in decision-making increases anxiety in cancer patients [4], [11]. Also decisional regret is not unusual in cancer patients [12]–[14]; patients who were less satisfied with their role in the decision-making process expressed more regret [12]. Yet, other studies have found that patient participation improved the decision-making process [15], [16] and a positive effect of patients' well-being [17], [18]. Demographic factors, such as age, sex, and education level, are associated with patients' preferences for information and involvement in disease management [19]–[21]. Most studies on patients' preferences for participation in treatment decision-making are conducted in the curative setting, where patients often have to choose between two treatments that have both proven to be effective, and where the advantages and disadvantages of both options are discussed and considered. For example, comparisons of mastectomy to breast-conserving therapy have demonstrated equivalent survival rates for women with early-stage breast cancer [22]. Less attention has been paid to shared decision-making in palliative care. When cure is no longer possible, treatments often might still prolong life, but with the possibility of side effects. Therefore, in treatment decision-making in palliative care, more emphasis is put on quality of life. A study among recurrent ovarian cancer patients found that they considered quality of life of secondary importance [23]. These women were willing to tolerate the toxicity of chemotherapy in the expectation of some life prolongation, regardless of the fact that the cancer was not curable. Studies in other patient groups, however, have shown that subjects preferred quality of life over quantity of life. Mack *et al*. [24] and Wright *et al*. [25] found that two thirds of advanced cancer patients preferred treatment focused on relieving pain and discomfort over life-extending treatment. Up to now, most studies on preferences for participation in treatment decision-making have focused primarily on determining the preferred level of involvement in treatment decision-making (active, shared or passive) [20], [21], [26], rather than exploring how patients interpret preferred levels of involvement and what reasons they have for their preference. A qualitative study among patients with colorectal cancer showed that patients preferred to be well informed and involved in the consultation process but did not necessarily want to make decisions, and the study found that the preferred level of participation depended on the type of decision that had to be made (treatment versus physical and psychological care) [27].

Little is known about patients' preferences for involvement in treatment decisions, in palliative care situations. The purpose of the present study was to obtain insight into patients' preferences and the reasons for patients' ideas of preferred role in treatment decision-making whether or not to start a life prolonging treatment in the near future.

## Methods

### Ethics Statement

The study was approved by the Medical Ethics Committee of the VU University Medical Center, Amsterdam. Besides, the participating departments (Neurology and Medical Oncology) gave their approval for the research to be carried out.

Written informed consent to participate in the study and publish the results was obtained of all respondents at each interview appointment.

### Design

We conducted a qualitative descriptive study [28] in which in-depth interviews with advanced cancer patients were performed.

### Study population

Two patient populations facing palliative treatment decisions were included in this study. The first group consisted of patients diagnosed with glioblastoma (GBM), the most common and most malignant type of primary brain tumor in adults, that underwent postoperative combined chemo- and radiotherapy [29]. These patients have a poor prognosis and cannot be cured of their disease. The median survival for these patients is approximately 14 months after diagnosis with current standard care [30].

The second patient population included in the study was a group of patients with metastatic colorectal cancer. Patients were eligible if they were diagnosed with metastatic colorectal cancer (stage IV) and were not eligible for operation. The median survival for these patients is 24-28 months with current standard care [31], and fewer than 5–8% of these patients are alive at five years from diagnosis [31], [32].The aims of chemotherapy in both patient populations are to prolong survival, control symptoms, and maintain or improve quality of life (e.g. relief of pain caused by tumor growth) [33]. Chemotherapy can be effective in prolonging time to disease progression and survival but these benefits must be weighed against treatment toxicity and the effect on quality of life (e.g. nausea and fatigue) [33].

In both patient groups, when progression of the disease occurs, a decision is often required on whether or not to start a (second-line) treatment aimed at prolonging life, but with the disadvantage of burdensome side effects.

Patients diagnosed with GBM were included at the beginning of their adjuvant temozolomide chemotherapy, soon after the end of the postoperative concomitant chemo-irradiation. Metastatic colorectal patients were included at the beginning of treatment with first-line palliative chemotherapy.

### Recruitment and inclusion

Patients were recruited in a large university hospital through consecutive sampling. The study with GBM patients started in May 2010 and patients were included till December 2012. Recruitment for colorectal cancer patients started in November 2011 and ended in February 2013. Patients were eligible if they were over the age of 18, spoke and understood Dutch, had been diagnosed with either GBM or metastatic colorectal cancer, and had started with first line treatment.

We considered all patient at the two departments that started with first line therapy during the inclusion period for our study. To identify the GBM patients the researcher (LB) attended multidisciplinary team meetings and two times a week a briefing where physicians prepared their out-patient clinic visits and patients' status and treatment plans were discussed. To identify metastatic colorectal patient the researcher attended a weekly briefing where physicians discussed new patients and their treatment plans. She also stayed in regular contact with physicians and nurses of the participating departments to identify eligible patients.

During the inclusion period we identified 47 patients with GBM and 11 patients with colorectal cancer possibly eligible for our study. Of these, 15 GBM patients were not eligible because they already had progression of the disease before the moment of inclusion. Furthermore, 2 GBM patients were not approached, because the physician thought the study would be too burdensome for them. This led to 30 GBM patient and 11 metastatic colorectal patients that were eligible and were approached for our study. They were handed an information letter by their physician during their visit on the outpatient clinic and after one week, the researcher (LB) phoned the patients and explained the study aims and methods to them. Of these patients 12 GBM patients and 1 metastatic colorectal patient declined to participate in the study of whom 5 patients were not interested in the study, 5 patients felt they were too ill to participate, 1 patient said it was too emotionally demanding because she had problems with her speech, 1 patient was too stressed about how the disease would develop in the future, and 1 patient did not want the researcher to attend patient-physician conversations.

This resulted in 28 participating patients (their characteristics are shown in Table 1), of whom 18 were diagnosed with GBM and 10 with metastatic colorectal cancer. The patients ranged in age from 27 to 82.

**Table 1:** Characteristics of participants

Participants	28
Men	18
Women	10
Age range	27–82
≤35	3
36–50	3
51–65	12
66–80	8
>81	2
Diagnosis	
Glioblastoma	18
Metastatic Colorectal cancer	10
CPS*	28
A	0
B	9
C	10
D	6
E	2

*1 patient was not able to pick a card

### Data collection

The in depth interviews lasted approximately 45–60 minutes and were held at the patients' homes. We used an interview topic list based on the objectives of the study. The list contained general questions about the patients' treatments so far; open-ended questions about their preferences for participation in future treatment decisions; communication with their treating physician and if they could imagine situation in which they would no longer want to receive treatment. The Control of Preferences Scale (CPS), developed by Degner [34], was used to start discussing the extent and type of influence patients wanted to have concerning treatment decision-making in the future. The CPS is a widely used tool to measure patient preferences for participation. The CPS is clinically relevant, easy to administer and valid in health care decision-making [34]. The CPS allowed the patient to choose one of five levels of involvement in decision-making, ranging from active to passive (Table 2). Further questions followed up on answers respondents provided and aimed to clarify why they had chosen a certain level of involvement. At the end of the interview the researcher checked whether all topics had been covered [35]. All interviews were performed by the first author (LB) and were recorded and transcribed verbatim. The interviewer kept field notes, describing her reflections on the interviews and the study.

**Table 2:** Control Preference Scale

A	I prefer to make the final decision about what treatment I will receive
B	I prefer to make the final selection of my treatment after seriously considering my doctor's opinion
C	I prefer that my doctor and I share responsibility for deciding which treatment is best for me
D	I prefer that my doctor makes the final decision about which treatment will be used, but seriously considers my opinion
E	I prefer to leave all decisions regarding my treatment to my doctor

During the interview all patients said that they understood the severity of their disease and were aware of the fact that at some point in the disease trajectory the disease would progress and new treatment decisions must be made. They also knew that they would eventually die from the disease. They told that their treating physician informed them on their diagnosis after their surgery (GBM patients) or after CT scans were made (metastatic colorectal patients).

### Data analysis

As our study was explorative, we used open coding as described by Strauss and Corbin [36]. Data analysis started during data collection and was an ongoing process. The constant comparative method was used to compare codes within and between interviews and to identify the main themes [37]. To ensure the reliability of the coding procedure, two researchers (LB and HRWP) independently read through the interviews to generate a list of codes (e.g. keeping control over own life, reaching consensus, shared responsibility, lack of knowledge, expertise physician and phase of illness), and compared their results. This revealed high agreement between the coders, and any disagreements were solved by discussion. The codes were discussed with two other researchers (with BDP and GAMW), and the group worked towards consensus about the interpretation of key themes. The main themes were identified and relevant quotes were chosen to illustrate these themes. The enrolment of participants was stopped once the analysis of the latest interviews did not generate new codes or enriching existing themes (theoretical saturation). A professional translator translated the quotes that we eventually chose to illustrate our results.

## Results

In exploring the preferred level of participation in upcoming treatment decision-making, almost all patients found it hard to pick the CPS card that best described their preferred role, and said they could imagine wanting different roles depending on the situation and type of decision. One patient could not make a final choice for the preferred level of participation for this reason. First, we will describe the CPS scores of patients and their reasoning for wanting that role in upcoming treatment decisions. After that, we focus on the factors patients mentioned that might influence or change their preferred role for participation in treatment decision-making.

### 1. Preferred participation and roles of patient and physician in upcoming treatment decision-making

Table 1 shows the patients' ultimate choice of CPS card regarding their role in upcoming treatment decisions whether or not to start a new palliative cancer treatment. All patients preferred the physician to have a role in the decision-making process. Two patients wanted their physician to decide for them in future treatment decisions (option E) and the other patients wanted a shared approach with varying degrees of influence from the physician (options B, C, or D).

All patients said that they wanted their physician to participate in the treatment decision-making process because of his/her knowledge and clinical experience with treatments and disease. The extent to which patients themselves wanted to participate in upcoming decisions seemed to be dependent on how patients saw their own role or how they assessed their own capabilities for participating in treatment decision-making. By balancing the different inputs from themselves and from the physician, patients came to a final perspective on the degree of influence they wanted to have. Patients who gave more responsibility to the physician than to themselves said that expertise was very important or thought their own knowledge was limited.

*R:“Yes, the doctor knows what's best for me. I can't judge that from a medical point of view. That's why I think the doctor should decide. […] Well, the actual decision is the doctor's, because medically speaking she.. I mean, I don't know anything about the subject. I haven't had any kind of medical training, so I don't know any of that kind of thing, so I just go by what the doctor says.”*

*[..]*

*R: “Yes, you may all think I want a say, but you don't have that knowledge, so what kind of a say do you have? You don't really have any say at all. All you can do is talk about how it all.. Yes, that's kind of what I mean, about what happens and what side effects you can expect and that you need to call if you feel something, that you discuss things like that with the doctors, that you know that. But well, I don't really think having a say applies at all in the case of cancer care.”*

***Woman, age range 51***–***65 years, metastatic colorectal cancer, CPS Card D***

Other patients attached more importance to reaching a consensus and having a shared responsibility in decision-making, for instance because they said they were the ones who knew themselves best.

*R: “In my opinion it's about sharing. And that applies to me too. You could say I'm the expert on the subject of me, and the doctor is the expert when it comes to the treatment and I reckon the two need to come together.”*

***Man, age range 36***–***50 years, GBM, CPS Card C***

*R: “It's not entirely true to say I take the final decision - you do it together. The two sides need to come to a kind of agreement, an intuitive one too. And then you subject yourself to it as a patient. Because look, of course you don't have all the know-how, most patients don't know anything at all. […] It's very difficult for most patients to get a good impression of what the treatment involves, what their clinical picture is, so of course that makes it quite a difficult decision. Sure, most people can still decide whether or not to subject themselves to it, of course, but as far as I'm concerned it's a case of taking a joint decision to go down that route, but definitely after discussing things together and after I've had a think about it.”*

***Man, age range 51***–***65 years, GBM, CPS Card C***

Several patients highly valued keeping control over their own lives and therefore said they wanted to have a more decisive role and the final say in upcoming treatment decisions. For example, one patient said:

*R: “And if it's not good, then I reckon they come to you, to us, with a suggestion. And then I reckon they come with a suggestion that I can say yes or no to, or can discuss the ins and outs, but I do want to take the final decision myself, including about the treatment.” […] R: “Yes, what does get done and what doesn't, whether to continue treatment or not, or the ins and outs. But I want to stay in control, that's really the main reason I chose B - no-one else decides this, just me.”*

***Man, age range 51***–***65 years, GBM, CPS Card B***

Other patients mentioned that they wanted to have the final say in the upcoming treatment decision because they felt it was their responsibility to make these decisions, or that they should not ‘bother' the physician with that:

*R:“ Look, basically I don't think you should dump the decision on the doctor. A doctor can say to me, well this is the situation, but you're the one who should take the final decision.”*

*I. “That's what you think…”*

*R: “Yes, I do think that, you can't do that to anybody. I'm convinced that the doctor says to me this isn't going to work out, or this is a good option, then I base it on his opinion, his view of things, and I am saying B because then I'm making the decision to follow your opinion.”*

*I: “I understand: so you‘re saying that in the end I'm responsible for that decision as the patient, I have to take it, but you do base it on the advice given by the doctors?”*

*R: “Yes of course. He's the cancer specialist, I'm a tiler.”[…] R: “Because ‘cobbler, stick to your last' may be an old saying but it's still true. But these are ethical questions and of course they are a lot more difficult. And I think you can't dump it on the doctor by saying well, I don't know what to do any more, why don't you decide. I think that… surely no doctor would do that, would they?”*

***Man, age range 51***–***65 years, metastatic colorectal cancer, CPS Card B***

### 2. Anticipated increase in participation

#### Illness trajectory

Most patients said that it was difficult to picture upcoming treatment decisions and, as stated above, a lot of patients found it hard to pick the CPS card that best described their preferred level of involvement in upcoming treatment decisions. They could imagine that the illness trajectory would influence their preferred level of participation and that their preference might change along with the phase of illness.

*R: “Well look, like I just said, at the moment I'm in this phase where I'm just managed by the doctors, as it were. And of course should it be the case in the future… that you end up in a hopeless situation, then I might say to myself why don't we just do this… or try something else, or as far as I'm concerned we stop, I no longer want it.” […] R: “Yes, and perhaps we start in this phase (D).. and then we go on to that phase (C) and then in the end I actually take a decision (B).”*

*R: “That's just how it is at the moment.. Well, I don't see any reason to take charge of making decisions.”*

***Man, age range 36***–***50 years, GBM, CPS Card B***

Several patients mentioned more specifically that their preferred role depended on the aim of the treatment, which would probably change along with the phase of the illness. If the prolongation of life were to remain one of the aims of both the physician and the patient, patients would want the physician to have a more decisive role because of his/her expertise and knowledge.

*R: “If he [the doctor] says it's a really good idea to do this, and it might make you sick now but it will let you keep going for another two years then I won't say ‘no, I don't want that'. Because it will give you another two years. […] But it's not that I want to make the decision myself, it's not that. Because if he really says it's better, it's good for you and you can do it.. yes, then I think to myself, well, you are trained in this. […] But if it's a case of it doesn't have any effect on how much longer you'll live, well, why should I do it then? […] Then I think no, I don't see the point in that.” **Woman, age range** ≤**35 years, GBM, CPS Card C***

But if the quality of life were to become more important, they would want to have a more decisive role themselves. They could imagine situations in which they might stop their initial treatment if it led to a substantial decline in their quality of life due to unacceptable treatment side effects or disease-related issues.

*R: “And if you were to end up in a situation where your condition is just not good and a treatment would really only mean stretching it out, or pain, or not feeling well, but not giving you much more benefit, then I can imagine you'd say ‘forget it'.”*

***Man, age range 36***–***50 years, GBM, CPS Card C***

One patient specifically said that if it came to a decision about life or death, she wanted to decide for herself:

*R2: “Yes. And also, we've really been talking about medical opinions all the time, of course lots of medical opinions are almost moral issues, aren't they: do you want to carry on living or not?”*

*I: “Yes.”*

*R2: “In the end that's not for the doctor to decide, is it?”*

*I: “No, but well.”*

*R2: “Doctors often take on that role but it's not for them to decide. And they shouldn't decide that either.”*

*R: “Well, the doctor will never do that as far as I'm concerned.”*

*I: “No.”*

*R: “Well of course.. Look, in a certain way the doctor does do that because it depends on how he explains what might happen. Of course you can always put it or tell that in such a way that it influences things. A doctor can't avoid doing that, ever. So of course he probably influences things to that extent. But ultimately, you are the one who decides about life and death.”*

***Woman, age range 51***–***65 years, GBM, not able to choose CPS card***

**(R2  =  patient's partner)**

#### Limits

Patients talked about limits they set for themselves to preserve their quality of life. Patients said they would decide to stop their initial treatment if that limit was reached, thus wanted to have a decisive role at that time.

*“And if after those three treatments I […] if you don't see any relief and I am getting sick, so not feeling as I do at the moment, then I don't know what I'll do, then I'll wait to hear what my cancer specialist says and I'll decide what I want to do on the basis of that. Because what I definitely don't want is for my children and my husband, first of all my husband and children and then my relatives and friends to have to come to my bedside to watch me fade away. No, I don't want that. No, that isn't … that wouldn't be an option for me, as I see it now.”*

***Woman, age range 51***–***65 years, metastatic colorectal cancer, CPS Card B***

Some mentioned clear limits, such as not ending in a vegetative state:

*“Just before the operation, when we discussed everything with the doctor, I said then: one thing for sure, I don't want to come out of this a vegetable. That was the one thing I was totally sure about and he was totally sure about too. So that wasn't even a question, a choice, for either of us. Not for the specialist and not for me.”*

***Woman, age range 51***–***65 years, GBM, not able to choose CPS card***

Many patients considered at the time of the interview that they had not reached their limits yet, and were still in the phase where they wanted to aim for life prolongation.

Several patients seemed unable to face or accept (a future) transition in aims from life prolongation to the quality of life, ending in death. For them, ‘doing nothing' was not an option because of their current good clinical condition, and therefore they felt they had no other choice than to start a treatment, because they wanted to postpone death:

*R: “You don't have a choice. You may say ‘I don't want treatment' but well, then.. especially in this situation, you don't have a choice. […] Well OK, no chemo, you don't get chemo as it were and then you just see what happens. But I think.. that's why you have so little choice in this situation.”*

*[…]*

*R: “Yes.. I think you would do an awful lot for your health. I would simply do anything. There has been no point up to now where I've thought well, I don't think that's admissible.”*

***Woman, age range 51***–***65, metastatic colorectal cancer, CPS Card D***

*R: “I love life and yes, I will take anything going and just try it.”*

*[…]*

*R: “And as far as this [the disease] is concerned, yes, perhaps it's an obstacle we can overcome. And perhaps I won't make it, but it doesn't matter, we'll go for it. Yes, 100%. Yes, of course. However bad I feel, I still think we have to keep going, old buddy, no complaining eh?”*

***Man, age range 66***–***80, metastatic colorectal cancer, CPS Card C***

## Discussion

Our results show that all patients preferred their physician to play a role in the decision-making process concerning whether or not to start a life-prolonging treatment because of his/her expertise and experience. The extent to which patients themselves wanted to participate seems to depend on how patients see their own role or assess their own capabilities for participating in upcoming treatment decision-making. Patients who doubted about their capacity to make treatment decisions by themselves preferred to give a more decisive role to their physician. But patients who highly valued keeping control over their own life wanted to have a more decisive role themselves, and patients who felt it was their responsibility to make treatment decisions thought that they ought to have a more active role themselves. However, patients also said that their role was not static and would probably change along with the illness trajectory. They imagined that their role would become more active as the disease progressed and quality of life would become more important.

### Variation in participation preferences

The finding that patients differ in their preference for participation in treatment decisions is found in other studies [38]–[40], as was the finding that patients prefer physicians to have some role in decision-making [41], [42]. The assumption that all patients want to play an active role in treatment decision-making is too simplistic, implying that more attention should be paid to individual wishes and needs.

Not only do patient preferences vary, the reasons for these preferences may also be different. We found that patients who preferred a more decisive role for themselves (option B) had different rationales for this choice: either they wanted to have control over their own life or they felt responsible for making the treatment decision themselves and not burdening the physician. In the first case, the patient wants to make a treatment decision for his or her own good, in the second, the patient acts out of consideration for the physician. This implies different support needs. In the first case, the patient needs adequate information and the freedom to choose; in the second case, the patient needs a shared view on mutual responsibilities. Thus, physicians should be open to the reasons behind a patient's preference and respond appropriately.

### Anticipated participation preferences related to treatment aim

Our finding that patients foresee a shift in the preferred level of participation from more passive to more active depending on the phase of the illness and the treatment aim is confirmed in other studies with patients who were already in the later stages of their illness trajectory: Sulmasy *et al*. [43] found that as terminally ill patients live longer with their disease, they prefer somewhat less reliance upon physicians, Cohen *et al*. found in a study of patients with prostate cancer [44] that the patients' willingness to become actively involved in choosing their care varied over time. Not only illness trajectories but also the type of decisions is found to be influential to the degree of participation. Beaver *et al*. [27] found that patient preferences depended on the type of decision as patients were more able to engage in decisions about the physical and psychological aspects of care than in treatment decisions.

The finding that patients' preferred role seems not to be static throughout the illness trajectory is important for physicians in daily practice, and they should take this into account when decisions have to be made during the illness trajectory. They should check whether the patient wants to change his or her role in decision-making as the disease progresses and palliative options become more prominent.

Patients' idea of having a more active role later on in the disease trajectory might not be easy and it can be questioned if it will happen. In the first place, patients may have difficulties in recognizing the stage of their disease. It is known that patients shift their limits and are willing to go on with burdensome treatments because they do not want to ‘give up' and face death [45]. Other studies found that patients want to hold on to their lives and are willing to accept intensive chemotherapy for a very small chance of benefit [46], [47].

In the second place, physicians seem to be inclined to offer further treatment when they are confronted with treatment dilemmas [48]. The mutually reinforcing attitudes of ‘not giving up' by patient and physician may lead to compromises such as “trying out one dose” [48]. Similar results were found by The *et al*. [49], who described a collusion between physician and patient that might hamper the shift from a life-prolonging aim towards a focus on the quality of life and could also prevent patients from taking the lead in the decision-making process. Our finding that patients felt they did not have a choice – that is, a choice between treatment and doing nothing – also shows that taking a more active role is not easy. Some patients felt there is no other choice than to go on with new treatments because the alternative – no treatment, which will lead to death – was not acceptable. Previous studies found similar results [50]–[52].

Furthermore, it is interesting to notice that apparently patients haven't perceived the choices until the moment of the interview as related to quality of life, although in all patients decisions with possible consequences for their quality of life were already made. For instance in GBM patients the decision to undergo surgery and to start with first line therapy and in metastatic colorectal cancer patients to start first-line treatment. We did not observe these decision-making processes, but we know from discussions with patients that these decisions were not even questioned by the physician and patient, as they wanted treatment anyhow and life prolongation was more important than quality of life at that moment.

Given that patients envisaged a more active role in treatment decision-making as the options for treatment would become more restricted, but also given that this role will probably not be taken automatically, we recommend that more attention be given to the careful planning of future treatment decisions, with an eye for adequate patient involvement in line with patient preferences. If treatment benefits are modest and discussed openly, the alternative option, of providing supportive care only, might be perceived as a reasonable alternative.

#### Strengths and limitations

A strength of our study is the qualitative approach, which provides an understanding of how patients see their own role in the treatment decision-making process at the end of life and offers further insight into the reasons behind treatment decisions. Also, this is one of the first studies that has been done on patients' preferences for participation in treatment decision-making in the advanced cancer setting. Our study was limited to patient preferences before actual treatment decision-making had taken place. It is possible that preferences change over time or that the perceived participation by patients differs from their preference. A recommendation for future research would be to follow patients over a longer period of time to see whether preferences do indeed change over time, with patients wanting more participation in later stages of their disease, and to get insight in the process of treatment decision-making. Another limitation is the lack of information on educational level of patients, since this can be relevant for preferred level of participation.

### Conclusion

Treatment decision-making in the last phase of life is often difficult and a highly complex process. Whereas all patients consider the role of the physician essential, there are differences between patients in their preferences for participation in treatment decision-making. In addition, individual patients' preferences may change in the course of the illness, with a shift to more active participation in the later phases. Whether this shift towards more patient-led decision-making is feasible in the near future is open to question. Communication about patients' expectations, wishes and preferences for participation in future treatment decisions is of great importance. An approach in which preferences, goals, and expectations are openly discussed would be beneficial. It could help fulfil patients' preferences in the treatment decision-making process and could help avoid situations in which opting to withhold life-prolonging treatment is perceived as doing nothing.

## References

[1] Blanchard CG, Labrecque MS, Ruckdeschel JC, Blanchard EB (1988) Information and decision-making preferences of hospitalized adult cancer patients. Social Science & Medicine 27: 1139–1145. 3206248 10.1016/0277-9536(88)90343-7

[2] Hack TF, Degner LF, Dyck DG (1994) Relationship between preferences for decisional control and illness information among women with breast cancer: a quantitative and qualitative analysis. Soc Sci Med 39: 279–289. 8066506 10.1016/0277-9536(94)90336-0

[3] Rothenbacher D, Lutz MP, Porzsolt F (1997) Treatment decisions in palliative cancer care: patients' preferences for involvement and doctors' knowledge about it. Eur J Cancer 33: 1184–1189. 9301440 10.1016/s0959-8049(97)00034-8

[4] Gattellari M, Butow PN, Tattersall MHN (2001) Sharing decisions in cancer care. Social Science & Medicine 52: 1865–1878. 11352412 10.1016/s0277-9536(00)00303-8

[5] Brown R, Butow P, Wilson-Genderson M, Bernhard J, Ribi K, et al (2012) Meeting the decision-making preferences of patients with breast cancer in oncology consultations: impact on decision-related outcomes. J Clin Oncol 30(8): 857–862. 22312102 10.1200/JCO.2011.37.7952

[6] Kremer H, Ironson G, Schneiderman N, Hautzinger M (2007) "It's my body'': does patient involvement in decision making reduce decisional conflict? Med Decis Making. 27: 522–532. 17873261 10.1177/0272989X07306782

[7] Purbrick RMJ, Tu KL, Damato BE (2006) The patient's role in the decision-making process—a perspective from the Liverpool Ocular Oncology Centre. Eye (Lond) 20: 1034–1039. 16138113 10.1038/sj.eye.6702074

[8] Sepucha KR, Ozanne EM, Partridge AH, Moy B (2009) Is there a role for decision aids in advanced breast cancer? Med Decis Making 29: 475–482. 19329775 10.1177/0272989X09333124

[9] Tariman JD, Berry DL, Cochrane B, Doorenbos A, Schepp K (2010) Preferred and actual participation roles during health care decision making in persons with cancer: a systematic review. Ann Oncol 21(6): 1145–1151. 19940010 10.1093/annonc/mdp534 PMC4200024

[10] Hubbard G, Kidd L, Donaghy E (2008) Preferences for involvement in treatment decision making of patients with cancer: A review of the literature. Eur J Oncol Nurs 12: 299–318. 18486552 10.1016/j.ejon.2008.03.004

[11] Levy SM, Herberman RB, Lee JK, Lippman ME, d'Angelo T (1989) Breast conservation versus mastectomy: distress sequelae as a function of choice. J Clin Oncol 7(3): 367–375. 2918332 10.1200/JCO.1989.7.3.367

[12] Clark JA, Wray NP, Ashton CM (2001) Living with treatment decisions: regrets and quality of life among men treated for metastatic prostate cancer. J Clin Oncol 19(1): 72–80. 11134197 10.1200/JCO.2001.19.1.72

[13] Clark JA, Inui TS, Silliman RA, Bokhour BG, Krasnow SH, et al (2003) Patients' perceptions of quality of life after treatment for early prostate cancer. J Clin Oncol 21(20): 3777–3784. 14551296 10.1200/JCO.2003.02.115

[14] Payne DK, Biggs C, Tran KN, Borgen PI, Massie MJ (2000) Women's regrets after bilateral prophylactic mastectomy. Ann Surg Oncol 7(2): 150–154. 10761795 10.1007/s10434-000-0150-6

[15] Brundage MD, Feldman-Stewart D, Dixon P, Gregg R, Youssef Y, et al (2000) A treatment trade-off based decision aid for patients with locally advanced non-small cell lung cancer. Health Expect 3(1): 55–68. 11281912 10.1046/j.1369-6513.2000.00083.x PMC5081084

[16] Fiset V, O'Connor AM, Evans W, Graham I, Degrasse C, et al (2000) Development and evaluation of a decision aid for patients with stage IV non-small cell lung cancer. Health Expect 3(2): 125–136. 11281919 10.1046/j.1369-6513.2000.00067.x PMC5080958

[17] Hack TF, Degner LF, Watson P, Sinha L (2006) Do patients benefit from participating in medical decision making? Longitudinal follow-up of women with breast cancer. Psychooncology 15: 9–19. 15669023 10.1002/pon.907

[18] Pozo C, Carver CS, Noriega V, Harris SD, Robinson DS, et al (1992) Effects of mastectomy versus lumpectomy on emotional adjustment to breast cancer: a prospective study of the first year postsurgery. J Clin Oncol 10(8): 1292–1298. 1634919 10.1200/JCO.1992.10.8.1292

[19] Arora NK, McHorney CA (2000) Patient preferences for medical decision making: who really wants to participate? Med Care 38(3): 335–341. 10718358 10.1097/00005650-200003000-00010

[20] Degner LF, Kristjanson LJ, Bowman D, Sloan JA, Carriere KC, et al (1997) Information needs and decisional preferences in women with breast cancer. JAMA 277(18): 1485–1492. 9145723

[21] Deber RB, Kraetschmer N, Urowitz S, Sharpe N (2007) Do people want to be autonomous patients? Preferred roles in treatment decision-making in several patient populations. Health Expect 10(3): 248–258. 17678513 10.1111/j.1369-7625.2007.00441.x PMC5060407

[22] Lantz PM, Janz NK, Fagerlin A, Schwartz K, Liu L, et al (2005) Satisfaction with Surgery Outcomes and the Decision Process in a Population-Based Sample of Women with Breast Cancer. Health Services Research 40: 745–767. 15960689 10.1111/j.1475-6773.2005.00383.x PMC1361166

[23] Donovan KA, Greene PG, Shuster JL, Partridge EE, Tucker DC (2002) Treatment preferences in recurrent ovarian cancer. Gynecol Oncol 86(2): 200–211. 12144829 10.1006/gyno.2002.6748

[24] Mack JW, Weeks JC, Wright AA, Block SD, Prigerson HG (2010) End-of-life discussions, goal attainment, and distress at the end of life: predictors and outcomes of receipt of care consistent with preferences. J Clin Oncol 28(7): 1203–1208. 20124172 10.1200/JCO.2009.25.4672 PMC2834470

[25] Wright AA, Mack JW, Kritek PA, Balboni TA, Massaro AF, et al (2010) Influence of patients' preferences and treatment site on cancer patients' end-of-life care. Cancer 116(19): 4656–4663. 20572030 10.1002/cncr.25217 PMC3670423

[26] Elkin EB, Kim SH, Casper ES, Kissane DW, Schrag D (2007) Desire for information and involvement in treatment decisions: elderly cancer patients' preferences and their physicians' perceptions. J Clin Oncol %20 25(33): 5275–5280. 18024875 10.1200/JCO.2007.11.1922

[27] Beaver K, Jones D, Susnerwala S, Craven O, Tomlinson M, et al (2005) Exploring the decision-making preferences of people with colorectal cancer. Health Expect 8(2): 103–113. 15860051 10.1111/j.1369-7625.2005.00320.x PMC5060279

[28] Sandelowski M (2000) Whatever happened to qualitative description? Res Nurs Health 23(4): 334–340. 10940958 10.1002/1098-240x(200008)23:4<334::aid-nur9>3.0.co;2-g

[29] Taphoorn MJ, Stupp R, Coens C, Osoba D, Kortmann R, et al (2005) Health-related quality of life in patients with glioblastoma: a randomised controlled trial. Lancet Oncol 6(12): 937–944. 16321761 10.1016/S1470-2045(05)70432-0

[30] Stupp R, Mason WP, van den Bent MJ, Weller M, Fisher B, et al (2005) Radiotherapy plus concomitant and adjuvant temozolomide for glioblastoma. N Engl J Med 352(10): 987–996. 15758009 10.1056/NEJMoa043330

[31] Chu E (2012) An update on the current and emerging targeted agents in metastatic colorectal cancer. Clin Colorectal Cancer 11(1): 1–13. 21752724 10.1016/j.clcc.2011.05.005

[32] Labianca R, Beretta GD, Kildani B, Milesi L, Merlin F, et al (2010) Colon cancer. Crit Rev Oncol Hematol 74(2): 106–133. 20138539 10.1016/j.critrevonc.2010.01.010

[33] Simmonds PC (2000) Palliative chemotherapy for advanced colorectal cancer: systematic review and meta-analysis. Colorectal Cancer Collaborative Group. BMJ 321(7260): 531–535. 10968812 10.1136/bmj.321.7260.531 PMC27466

[34] Degner LF, Sloan JA, Venkatesh P (1997) The Control Preferences Scale. Can J Nurs Res 29(3): 21–43. 9505581

[35] Britten N (1995) Qualitative interviews in medical research. BMJ 311(6999): 251–253. 7627048 10.1136/bmj.311.6999.251 PMC2550292

[36] Strauss A, Corbin J (1990) Basics of qualitative research; Techniques and Procedures for Developing Grounded Theory. Sage.

[37] Glaser BG, Strauss AL (1967) The Discovery of Grounded Theory: Strategies for Qualitative Research. Aldine Publishing Company, Chicago.

[38] Berendsen AJ, de Jong GM, Schuling J, Bosveld HE, de Waal MW, et al (2010) Patient's need for choice and information across the interface between primary and secondary care: a survey. Patient Educ Couns 79(1): 100–105. 19713065 10.1016/j.pec.2009.07.032

[39] Caress AL, Beaver K, Luker K, Campbell M, Woodcock A (2005) Involvement in treatment decisions: what do adults with asthma want and what do they get? Results of a cross sectional survey. Thorax 60: 199–205. 15741435 10.1136/thx.2004.029041 PMC1747348

[40] Janz NK, Wren PA, Copeland LA, Lowery JC, Goldfarb SL, et al (2004) Patient-physician concordance: preferences, perceptions, and factors influencing the breast cancer surgical decision. J Clin Oncol 22(15): 3091–3098. 15284259 10.1200/JCO.2004.09.069

[41] Beaver K, Booth K (2007) Information needs and decision-making preferences: comparing findings for gynaecological, breast and colorectal cancer. Eur J Oncol Nurs 11: 409–416. 17604693 10.1016/j.ejon.2007.04.004

[42] Carey M, Anderson A, Sanson-Fisher R, Lynagh M, Paul C, et al (2012) How well are we meeting haematological cancer survivors' preferences for involvement in treatment decision making? Patient Educ Couns 88: 87–92. 22296772 10.1016/j.pec.2011.12.014

[43] Sulmasy DP, Hughes MT, Thompson RE, Astrow AB, Terry PB, et al (2007) How would terminally ill patients have others make decisions for them in the event of decisional incapacity? A longitudinal study. J Am Geriatr Soc 55(12): 1981–1988. 18031490 10.1111/j.1532-5415.2007.01473.x PMC2583169

[44] Cohen H, Britten N (2003) Who decides about prostate cancer treatment? A qualitative study. Fam Pract 20(6): 724–729. 14701899 10.1093/fampra/cmg617

[45] Fried TR, Bradley EH (2003) What matters to seriously ill older persons making end-of-life treatment decisions?: A qualitative study. J Palliat Med 6(2): 237–244. 12854940 10.1089/109662103764978489

[46] McQuellon RP, Muss HB, Hoffman SL, Russell G, Craven B, et al (1995) Patient preferences for treatment of metastatic breast cancer: a study of women with early-stage breast cancer. J Clin Oncol 13(4): 858–868. 7707112 10.1200/JCO.1995.13.4.858

[47] Slevin ML, Stubbs L, Plant HJ, Wilson P, Gregory WM, et al (1990) Attitudes to chemotherapy: comparing views of patients with cancer with those of doctors, nurses, and general public. BMJ 300(6737): 1458–1460. 2379006 10.1136/bmj.300.6737.1458 PMC1663147

[48] Buiting HM, Rurup ML, Wijsbek H, van ZL, den HG (2011) Understanding provision of chemotherapy to patients with end stage cancer: qualitative interview study. BMJ 342: d1933. 21464103 10.1136/bmj.d1933 PMC3070432

[49] The AM, Hak T, Koeter G, van Der WG (2000) Collusion in doctor-patient communication about imminent death: an ethnographic study. BMJ 321(7273): 1376–1381. 11099281 10.1136/bmj.321.7273.1376 PMC27539

[50] Elit L, Charles C, Dimitry S, Tedford-Gold S, Gafni A, et al (2010) It's a choice to move forward: women's perceptions about treatment decision making in recurrent ovarian cancer. Psychooncology 19(3): 318–325. 19319830 10.1002/pon.1562

[51] Pentz RD, Pelletier W, Alderfer MA, Stegenga K, Fairclough DL, et al (2012) Shared decision-making in pediatric allogeneic blood and marrow transplantation: what if there is no decision to make? Oncologist 17(6): 881–885. 22615217 10.1634/theoncologist.2011-0446 PMC3380888

[52] Weeks JC, Catalano PJ, Cronin A, Finkelman MD, Mack JW, et al (2012) Patients' expectations about effects of chemotherapy for advanced cancer. N Engl J Med 367(17): 1616–1625. 23094723 10.1056/NEJMoa1204410 PMC3613151

